# Choice of Growth Standard and Interpretation of Large for Gestational Age Outcomes: Evidence From the CDC4G Trial

**DOI:** 10.1111/ppe.70180

**Published:** 2026-07-29

**Authors:** Sara Grass, Maryam de Brun, Anders Magnuson, Martina Persson, Anna‐Karin Wikström, David Simmons, Snehal Patil, Verena Sengpiel, Scott Montgomery, Kerstin Berntorp, Ulla‐Britt Wennerholm, Helena Strevens, Karin Hildén, Elisabeth Storck‐Lindholm, Helena Backman, Fredrik Ahlsson

**Affiliations:** ^1^ Department of Women's and Children's Health Uppsala University Uppsala Sweden; ^2^ Astrid Lindgren Children's Hospital Karolinska University Hospital Stockholm Sweden; ^3^ Department of Obstetrics and Gynecology, Faculty of Medicine and Health Örebro University Örebro Sweden; ^4^ Clinical Epidemiology and Biostatistics, School of Medical Sciences, Faculty of Medicine and Health Örebro University Örebro Sweden; ^5^ Department of Clinical Science and Education Karolinska Institutet Stockholm Sweden; ^6^ Department of Medicine, Clinical Epidemiology Karolinska Institutet Stockholm Sweden; ^7^ Sachsska Childrens' and Youth Hospital Stockholm Sweden; ^8^ Akademiska sjukhuset Uppsala Sweden; ^9^ School of Medical Science, Faculty of Medicine and Health Örebro University Örebro Sweden; ^10^ Macarthur Clinical School Western Sydney University Campbelltown Australia; ^11^ Department of Obstetrics and Gynecology, Institute of Clinical Science, Sahlgrenska Academy University of Gothenburg Gothenburg Sweden; ^12^ Department of Obstetrics and Gynecology Sahlgrenska University Hospital, Region Västra Götaland Gothenburg Sweden; ^13^ Clinical Epidemiology Division, Department of Medicine, Solna Karolinska Institutet Stockholm Sweden; ^14^ Department of Epidemiology and Public Health University College London UK; ^15^ Genetics and Diabetes Research Unit, Department of Clinical Sciences Malmö Lund University Malmö Sweden; ^16^ Department of Obstetrics and Gynecology, Skåne University Hospital, Department of Clinical Sciences Lund Lund University Lund Sweden; ^17^ Department of Obstetrics and Gynecology Södersjukhuset Karolinska Institute Sweden

**Keywords:** gestational diabetes mellitus, growth charts, large for gestational age

## Abstract

**Background:**

The lack of a standardised definition of large for gestational age (LGA) complicates clinical assessments and the interpretation of research.

**Objective:**

The objective of this study is to assess how different definitions of LGA affect the interpretation of outcomes in a stepped wedge cluster randomised controlled trial (SW‐CRT).

**Methods:**

Secondary analysis of the Changing Diagnostic Criteria for Gestational Diabetes (CDC4G, ISRCTN41918550) study population (*n* = 47,080 pregnancies), including eight delivery units in Sweden during 2018. The outcome measures were the prevalence of LGA and severe LGA before and after switching from the previous Swedish diagnostic criteria for gestational diabetes (SWE‐GDM) to the World Health Organisation (WHO‐2013) criteria. LGA and severe LGA were defined as birthweight > 90th and > 97th percentile, respectively, using the following reference growth charts: The new Swedish reference ranges for fetal weight (SWE‐REF), the Gestation Related Optimal Weight (GROW) and the International Fetal and Newborn Growth Consortium for the 21st Century (Intergrowth‐21st). Analyses were performed on a modified intention‐to‐treat (mITT) population and on the subgroup discordant for GDM diagnosis between new and former criteria, i.e., glucose levels between the WHO‐2013 and SWE‐GDM diagnostic thresholds.

**Results:**

In the mITT population, the prevalence of LGA decreased from 19.4% to 13.1% after the switch to the WHO‐2013 GDM criteria, as defined by SWE‐REF (adjusted relative risk [RR], 0.92; 95% confidence interval [CI] 0.86, 0.98). There was no change in severe LGA using any of the reference charts. In the subgroup, LGA defined by SWE‐REF (RR 0.68, 95% CI 0.50, 0.91) and GROW (RR 0.78, 95% CI 0.63, 0.95) was reduced, but not when defined by Intergrowth‐21st (RR 0.88, 95% CI 0.74, 1.06). Severe LGA was reduced only when defined by Intergrowth‐21st (RR 0.73, 95% CI 0.62, 0.85).

**Conclusions:**

The choice of LGA definition affected the interpretation of the results in the CDC4G trial.

## 1 Introduction

The term large‐for‐gestational‐age (LGA) infants is widely used in research and clinical practice and is associated with maternal and neonatal complications [1, 2]. LGA is a recommended outcome variable in research evaluating interventions related to gestational diabetes (GDM) [3]. A systematic review conducted by The International Association of Diabetes and Pregnancy Study Groups (IADPSG) demonstrated that the definitions of LGA used in randomised controlled trials (RCT) vary considerably [4]. Some LGA definitions are insufficiently sensitive to identify some adverse outcomes, and most do not account for variations in ethnicity. LGA is usually defined as birthweight above the 90th percentile for gestational age and sex in a reference population, though stricter thresholds (95th or 97th percentile) have been suggested to better identify infants at risk of morbidity and mortality [5, 6, 7]. Various reference growth charts are used, with substantial methodological heterogeneity. There is no internationally agreed standardised definition of LGA, and the various methodologies do not differentiate between excessive and constitutional fetal growth. This is important because, by definition, the choice of a 90th percentile cutoff will incorrectly classify a large number of constitutionally larger infants as abnormal, even though their size is within the normal range and not associated with increased risk of morbidity.

A universally agreed reference growth chart in research could facilitate cross‐population comparisons, but birthweight distributions vary with ethnicity and maternal characteristics such as height, weight and parity [8, 9, 10, 11]. In recent RCTs evaluating GDM treatment, in which LGA has been the main or secondary outcome, the treatment effect has varied [12, 13, 14]. The trials have used different growth charts and are performed in different populations, making comparisons difficult. A review of guidelines for fetal growth restriction and small for gestational age (SGA) found that four out of six recommended customised reference charts, and all guidelines emphasised accurate gestational age assessment [15].

The Changing Diagnostic Criteria for Gestational Diabetes (CDC4G) trial examined the impact of changing from the former Swedish diagnostic criteria for GDM (SWE‐GDM) to the World Health Organisation 2013 diagnostic criteria (WHO‐2013) for GDM in Sweden, which has a lower diagnostic threshold [12]. The primary outcome was LGA based on the Swedish Maršál et al. growth charts [16] using the most common definition of birthweight > 90th percentile for gestational age and sex. A predefined decision was made to use this definition of LGA in the trial. The introduction of the WHO‐2013 GDM criteria did not affect LGA risk using the chosen definition. However, the incidence of LGA, defined as > + 2 standard deviations (SD) (approximately > 97th percentile) using the same charts, which is the clinically used definition in Sweden, was reduced. Due to difficulties in the interpretation of treatment trials for GDM, which is partly a reflection of the choice of growth charts, the aim of this secondary analysis of the CDC4G trial was to assess how the use of different growth charts and percentile thresholds for the definition of LGA affects the interpretation of whether treatment for GDM is beneficial or not.

## 2 Methods

### 2.1 CDC4G Trial and Study Population

Data from the CDC4G trial (ISRCTN41918550) were used to conduct this study. The CDC4G trial was a national prospective, unblind, stepped wedge cluster randomised controlled trial (SW‐CRT) of the switch from the SWE‐GDM diagnostic criteria (control group) to the WHO‐2013 criteria [17] for GDM (intervention group). The trial was conducted in Sweden in 2018, with the last birth in August 2019. The methods and results have previously been published [12, 18]. During the study period, referral for a diagnostic 75‐g oral glucose tolerance test (OGTT) was based on clinical risk factors, repeated measurements of random plasma glucose, and clinical indications. The SWE‐GDM criteria across regions have predominantly been those of overt diabetes, a fasting plasma glucose value ≥ 7.0 mmol/L, and/or a 2‐h value between 8.9 and ≥ 11.1 mmol/L. After the switch to the WHO‐2013 criteria, a 3‐point OGTT was introduced, with fasting plasma glucose and 1‐h and 2‐h diagnostic thresholds of ≥ 5.1, ≥ 10.0, and/or ≥ 8.5 mmol/L, respectively.

All pregnant women who received maternal healthcare within the participating regions in Sweden during the study period, and their offspring from that pregnancy, were included in the CDC4G study. Women with pre‐existing diabetes, history of gastric bypass surgery, multifetal pregnancies and those who chose to opt out of the Swedish Pregnancy Register (SPR) were excluded. In 2018 and 2019, 98.5% and 97.9% of all pregnancies in Sweden, respectively, were included in the SPR [19]. Data were collected from national health and quality registers [12] and through an electronic case report form (e‐CRF) with data manually entered from medical journals. The data was de‐identified and linked together by the Swedish National Board of Health and Welfare (SNBHW). The primary analyses were conducted according to the modified intention‐to‐treat principle (mITT) [20], excluding pregnancies within clusters that were randomised but excluded before the intervention. The mITT population consisted of 47,080 pregnancies. Secondary analyses were conducted on the subgroup of 2195 women who were discordant for GDM diagnosis. These had glucose concentrations between the diagnostic thresholds of the WHO‐2013 and SWE‐GDM criteria, i.e., fasting glucose 5.1–6.9 mmol/L and/or 2‐h glucose 8.5–8.9 mmol/L when tested for GDM with an OGTT. This means that they were diagnosed with and received treatment for GDM in the intervention group, but not in the control group.

### 2.2 Outcome

The predefined outcome of this study was the proportion of LGA births before and after the switch in diagnostic criteria for GDM, using different reference growth charts and percentile thresholds to define LGA, with the aim of comparing how different definitions of LGA affect the interpretation of results. LGA was defined as birthweights above the 90th percentile, and severe LGA as above the 97th percentile (approximately +2 SD) relative to the mean weight for gestational age and sex in each reference population. The reference growth charts included were three distinct reference growth charts: the new Swedish reference ranges for fetal weight (SWE‐REF) [21], the customised Gestation Related Optimal Weight (GROW) calculator [22], and the International Fetal and Newborn Growth Consortium for the 21st Century (Intergrowth‐21st) [23]. The results were compared with the those in the CDC4G main study, in which Maršál et al. [16] growth charts were used to define LGA.

### 2.3 Growth Charts

The growth charts currently used in Sweden by Maršál et al. [16] were published in 1996 and are based on fetal measurements estimated by ultrasound. Their applicability has been questioned as they are based on a small sample (*n* = 86) of uncomplicated pregnancies with birth at term, and 24% of the pregnant women were smokers. Lindström et al. recently presented new Swedish reference ranges for fetal weight (SWE‐REF) [21] based on a prospective longitudinal, multicentre study of 583 healthy, non‐smoking women with low‐risk pregnancies and full‐term births. Women with gestational hypertension, pre‐eclampsia or eclampsia, gestational diabetes, placenta previa, fetal growth restriction, malformations, chromosomal aberrations or stillbirth were excluded. Additional intrauterine biometrics from ultrasonography were used. These reference ranges are not yet used in clinical practice. The widely used customised GROW calculator is based on birthweights and maternal characteristics from over 4 million international full‐term pregnancies [22]. An individual optimal weight for gestational week 40 + 0 is calculated by adjusting for maternal characteristics known to affect fetal growth. Hadlock's expected fetal weight curve [24] is then used to calculate optimal weights for each gestational age. Furthermore, the International Fetal and Newborn Growth Consortium for the 21st Century (Intergrowth‐21st) has developed newborn weight standards intended for universal use across populations. They are based on anthropometric data at birth from 20,486 newborns in eight countries [23]. The characteristics of the different growth charts are listed in more detail in Table 1.

**TABLE 1:** Characteristics of the Reference Growth Charts.

Growth chart	Population and setting	Measurements and adjustments
SWE‐REF [21]	Prospective longitudinal multicentre study. Swedish population, 583 healthy pregnant women with low risk of abnormal fetal growth. 2015–2018. No maternal BMI limits.	Fetal biometrics (BPD, HC, AD, AC and FL) measured by sonography five times between GW 12 + 3 and 41 + 6. Fetal weight was estimated using Hadlock's formula. Adjusted for neonatal sex and gestational age. GA was confirmed by ultrasound measuring BPD at the first visit (GW 12 + 3–13 + 6 according to last menstrual period), and GA deviating > 7 days were excluded.
GROW [22]	Anonymised international database of over 4 million full‐term births from over 30 countries, including information on over 100 ethnic groups or countries of origin.	An individual term optimal weight (TOW) at 40 + 0 weeks gestation is calculated based on birthweight adjusted for maternal height and weight, ethnicity, parity, neonatal sex and gestational age. A customised fetal weight curve for each gestational age is then calculated by a model (33) based on Hadlock's EFW formula [24], but expressed as percentages of TOW. GA was determined by local guidelines.
Intergrowth‐21st [23]	Prospective, longitudinal, population‐based study. Brazil, Italy, Oman, UK, USA, China, India and Kenya, 2009–2013, 20,486 pregnancies with low risk of impaired fetal growth.	Anthropometrics (weight, length and HC) measured within 12 h of birth. Adjusted for neonatal sex and gestational age. GA was determined by sonography using CRL before GW 14 or BPD if antenatal care started between GW 14 and 24.
Maršál et al. [16]^a^	Prospective, longitudinal study of 86 women with uncomplicated pregnancies, 24% smokers. Four centres in Sweden and Denmark.	Fetal biometrics (BPD, AD and FL) measured by sonography 3–4 times per foetus in one centre and 7–11 times in the other three centres, between GW 27 + 1–40 + 0 and 10 + 6–40 + 6, respectively. Adjusted for neonatal sex and gestational age. GA was confirmed by ultrasound measuring BPD in the early second trimester (*n* = 72) or CRL in the first trimester (*n* = 14) and never deviated > 7 days from GA according to last menstrual period.

Abbreviations: AC, Abdominal Circumference; AD, Abdominal Diameter; BPD, Biparietal Diameter; CRL, Crown‐Rump Length; EFW, Estimated Fetal Weight; FL, Femur Length; GA, Gestational Age; GROW, Gestation Related Optimal Weight; GW, Gestational Week; HC, Head Circumference; Intergrowth‐21st, International Fetal and Newborn Growth Consortium for the 21st Century; OFD, Occipitofrontal Diameter.

^a^ Used in the CDC4G main study [12].

### 2.4 Statistical Analysis

Sample size calculation and analyses were conducted according to recommendations for SW‐CRT [25, 26]. Relative risks (RRs) with 95% confidence intervals (CIs) were calculated using multilevel mixed Poisson regression with robust standard errors, comparing outcomes between the WHO‐2013 and SWE‐GDM criteria groups, with clusters as random effects and period (January–March, April–June, July–September, October–December) as fixed effects (adjusted 1^a^). Adjustments were then made for potential confounding variables for LGA (maternal age at delivery, parity, chronic hypertension, smoking or snuff use in early pregnancy, and country of birth) (adjusted 2^b^). Maternal age was modelled by linear, squared, and cubic terms. Analyses were conducted in the same way for each reference growth chart, using both 90th and 97th percentile thresholds. The statistical analyses were performed according to the modified intention‐to‐treat (mITT) principle, described earlier. The same analyses were performed on the subgroup discordant for the diagnosis of GDM. A sensitivity analysis restricted to term births (gestational age ≥ 37 + 0 weeks) was conducted, using the same analytical approach, to assess whether the results remained consistent.

Analyses were performed in STATA (StataCorp. 2021. Stata Statistical Software: Release 17. College Station, TX: StataCorp LLC).

### 2.5 Missing Data

Multiple imputation by chained equations was used in the CDC4G main study [12] to impute 10 data sets for missing confounding variables. The imputation model included all explanatory variables noted above, as well as maternal BMI at first visit, educational level, and clusters. The STATA command *mi estimate* was used to adjust for variability between the imputed data sets according to Rubin's rules.

### 2.6 Ethics Approval

The Uppsala‐Örebro Regional Ethical Review Board (2016/487) and the Swedish Ethical Review Authority (2019/02148, 2020/02856, 2021/02055, 2021–03405) gave ethical approval to the CDC4G study.

## 3 Results

The CDC4G trial included 24,283 pregnancies in the intervention group (the WHO‐2013 criteria period) and 22,797 in the control group (the SWE‐GDM criteria period) in the mITT population. A total of 3135 OGTTs were performed using the SWE‐GDM criteria, and 3747 were performed using the WHO‐2013 criteria. The prevalence of GDM in the mITT population increased from 2.6% to 6.6% after the switch to the WHO‐2013 criteria. These results and the baseline characteristics have been previously published in the CDC4G study [12], but for clarification, baseline characteristics are also shown in Table S1.

### 3.1 Large for Gestational Age

The LGA prevalence varied depending on the reference growth chart and percentile threshold used, as shown in Table 2. The overlap between infants classified as LGA across the different reference charts in the entire CDC4G study population is illustrated in Figure 1. A total of 12,902 infants (27.4%) were classified as LGA by at least one of the included reference growth charts. Among these, only 2239 infants (17.4%) were classified as LGA across all three growth charts. There was a reduction in the incidence of LGA (> 90th percentile) in the mITT population after the switch to the WHO‐2013 GDM criteria, as defined by the SWE‐REF ranges. There was no decrease in LGA (> 90th percentile) with either the Intergrowth‐21st or GROW reference charts. No change in the incidence of severe LGA (> 97th percentile) was observed in the mITT group across the SWE‐REF, Intergrowth‐21st, or GROW reference growth charts.

**TABLE 2:** Outcomes in the Modified Intention to Treat and in the Subgroup Discordant for Diagnosis of GDM^a^.

Modified intention to treat						
	WHO‐2013 GDM criteria group (*n* = 24,283)		SWE‐GDM criteria group (*n* = 22,797)		WHO‐2013 vs. SWE‐GDM criteria group	
	n	Outcomes (%)	n	Outcomes (%)	Adjusted 1^b^ RR (95% CI)	Adjusted 2^c^ RR (95% CI) [1]
LGA						
LGA 90th perc SWE REF	24,209	1323 (5.5)	22,707	1248 (5.5)	0.91 (0.84, 0.98)	0.92 (0.86, 0.98)
LGA 90th perc, GROW	24,203	2281 (9.4)	22,704	2204 (9.7)	0.98 (0.93, 1.04)	0.98 (0.91, 1.04)
LGA 90th perc, Intergrowth‐21st	24,001	5882 (24.5)	22,549	5518 (24.5)	0.99 (0.95, 1.04)	1.00 (0.95, 1.05)
Severe LGA						
LGA 97th perc, SWE REF	24,209	403 (1.7)	22,707	360 (1.6)	0.93 (0.82, 1.05)	0.93 (0.81, 1.07)
LGA 97th perc, GROW	24,203	900 (3.7)	22,704	833 (3.7)	0.98 (0.90, 1.06)	0.96 (0.87, 1.07)
LGA 97th perc, Intergrowth‐21st	24,001	2280 (9.5)	22,549	2153 (9.5)	0.93 (0.87, 0.99)	0.94 (0.86, 1.01)
Results from the CDC4G main study^d^						
LGA 90th perc, Maršál et al.	24,209	2790 (11.5)	22,707	2584 (11.4)	0.96 (0.92, 1.01)	0.97 (0.91, 1.02)
LGA 2 SD, Maršál et al.	24,209	1065 (4.4)	22,707	1019 (4.5)	0.90 (0.83, 0.99)	0.91 (0.84, 0.99)

Subgroup discordant for GDM diagnosis^a^						
	WHO‐2013 GDM criteria group (*n* = 1239)		SWE‐GDM criteria group (*n* = 956)		WHO‐2013 vs. SWE‐GDM criteria group	
	n	Outcomes (%)	n	Outcomes (%)	Adjusted 1^b^ RR (95% CI)	Adjusted 2^c^ RR (95% CI)
LGA						
LGA 90th perc, SWE REF	1237	162 (13.1)	947	184 (19.4)	0.63 (0.47, 0.85)	0.68 (0.50, 0.91)
LGA 90th perc, GROW	1237	184 (14.9)	946	202 (21.4)	0.79 (0.64, 0.99)	0.78 (0.63, 0.95)
LGA 90th perc, Intergrowth‐21st	1229	435 (35.4)	944	417 (44.2)	0.85 (0.71, 1.03)	0.88 (0.74, 1.06)
Severe LGA						
LGA 97th perc, SWE REF	1237	69 (5.6)	947	61 (6.4)	0.74 (0.42, 1.31)	0.76 (0.36, 1.60)
LGA 97th perc, GROW	1237	95 (7.7)	946	99 (10.5)	0.93 (0.53, 1.62)	0.87 (0.55, 1.39)
LGA 97th perc, Intergrowth‐21st	1229	211 (17.2)	944	238 (25.2)	0.69 (0.59, 0.80)	0.73 (0.62, 0.85)
Results from the CDC4G main study^d^						
LGA 90th perc, Maršál et al.	1237	278 (22.5)	947	273 (28.8)	0.83 (0.71, 0.97)	0.87 (0.75, 1.01)
LGA 2 SD, Maršál et al.	1237	141 (11.4)	947	157 (16.6)	0.73 (0.57, 0.93)	0.77 (0.62, 0.96)

Abbreviations: CI, Confidence interval; GDM, gestational diabetes mellitus; Perc, Percentile; RR, Relative risk.

^a^ The cohort of women with fasting and 2‐h plasma glucose cut off between the WHO‐2013 criteria and SWE‐GDM criteria (fasting plasma glucose 5.1–6.9 and/or 2‐h plasma glucose 8.5–8.8/8.9/9.9 mmol/L), untreated before and treated after the switch.

^b^ Analysed with multilevel mixed Poisson model with cluster as random factor and period (January–March, April–June, July–September, October–December) as fixed factor.

^c^ Adjusted also for mother's age modelled by a linear, squared, and cubic term, chronic hypertension, smoking, snuff, country of birth, and parity, and multiple imputation used for missing potential confounding variables.

^d^ Results from the CDC4G main study, included for comparison [12].

**FIGURE 1:**
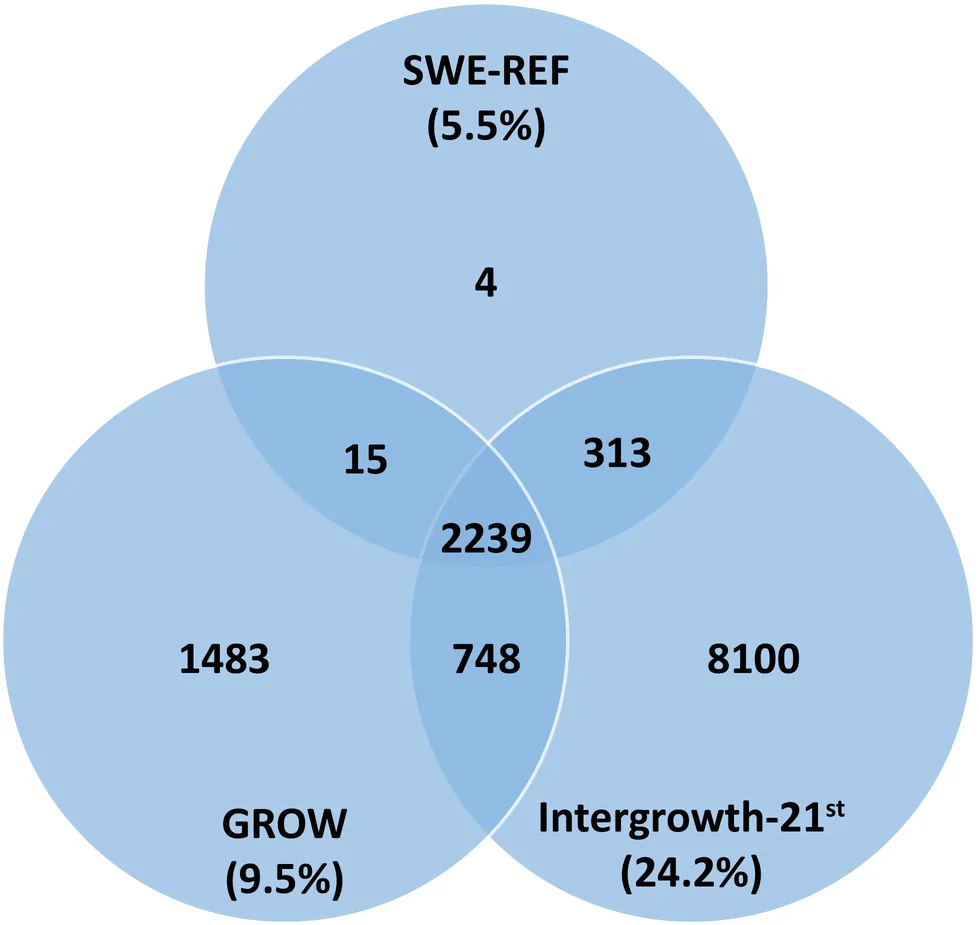
Overlap of infants defined as LGA (> 90th Percentile) according to SWE‐REF, GROW and Intergrowth‐21st and Prevalence in the entire modified intention to treat study population (*n* = 47,080).

In the subgroup of women discordant for GDM diagnosis, a greater reduction in the incidence of LGA (> 90th percentile) and severe LGA (> 97th percentile) was observed after the switch to the WHO‐2013 criteria, compared with the mITT population. When using the SWE‐REF ranges, the prevalence of LGA decreased from 19.4% to 13.1% (aRR 0.68, 95% CI 0.50, 0.91), but there was no change in severe LGA (> 97th percentile). When the GROW reference chart was applied, LGA (> 90th percentile) decreased from 21.4% to 14.9% after the switch (aRR 0.78, 95% CI 0.63, 0.95), but there was no reduction in severe LGA (> 97th percentile). According to the Intergrowth‐21st reference chart, there was no decrease in LGA (> 90th percentile). However, there was a reduction of severe LGA (> 97th percentile) from 25.2% before to 17.2% after the switch, aRR 0.73 (95% CI 0.62–0.85). The results from the CDC4G main study, using Maršál et al. growth charts, are included in Table 2 for comparison.

### 3.2 Sensitivity Analysis

In the sensitivity analysis restricted to term births, the results remained consistent, except for LGA defined by the Maršál et al. > 90th percentile, which decreased in the subgroup discordant for GDM diagnosis. The results of the sensitivity analysis are shown in Table S2.

## 4 Comment

### 4.1 Principal Findings

This study demonstrates that the choice of growth chart and percentile threshold for defining LGA affects the interpretation of whether changing to the WHO‐2013 diagnostic criteria for GDM reduced the risk of LGA births in Sweden. The number of infants categorised as LGA varied widely across the growth charts and, as expected, the percentage reduction in LGA was generally greater in the subgroup discordant for GDM diagnosis.

### 4.2 Strengths of the Study

Strengths of this study include the large, population‐based cohort with prospectively collected data in the CDC4G trial, which used a SW‐CRT design. Swedish national quality registers are comprehensive, with coverage exceeding 95% [19], thereby minimising the risk of selection bias in the CDC4G trial. Additional data were collected via an eCRF, with quality checks of OGTT blood glucose values. Thus, the study population is considered representative of pregnant women in Sweden.

### 4.3 Limitations of the Study

A potential limitation of this study is the modest number of growth charts included. However, the selected growth charts were carefully chosen and prespecified in the CDC4G study protocol, and the inclusion of additional charts would not likely affect the main conclusions of the study. In the subgroup analysis, no comparison could be made based on the one‐hour OGTT value, as mask one‐hour OGTT values were not implemented in clinical practice at any of the clusters before the switch to the WHO‐2013 criteria.

### 4.4 Interpretation

Suspected or excessive fetal growth leads to several clinical interventions to decrease the risk of maternal and neonatal complications, including induced labour or elective caesarean section [27]. Vaginal deliveries are monitored more closely, as LGA births have a higher risk of birth complications such as shoulder dystocia, brachial plexus injuries and postpartum haemorrhage [1]. After birth, LGA infants often receive supplementary feeding and are monitored for hypoglycaemia [28], and their mothers are more likely to be screened for GDM in future pregnancies if universal screening is not in use. Since both suspected and at birth LGA diagnoses often lead to clinical interventions, the definitions used in clinical practice will also affect several other outcomes and research results, thereby impacting clinical routines. Correct identification of excessive intrauterine growth, which indicates an increased risk of morbidity, is important for improving prevention, risk assessment, efficiency, and health benefits.

The optimal definition of LGA likely differs across populations due to variations in maternal characteristics and genetic factors. Therefore, it is important to report maternal characteristics in clinical trials. The WHO‐2013 diagnostic criteria are based on a multinational study reporting an association between hyperglycaemia in pregnancy, below the previously used cut‐off values for GDM, and increased risk of adverse outcomes [29]. In that study, LGA was defined as birthweight at or above the 90th percentile within the cohort, adjusted for gestational age, sex, parity, ethnic group, and study centre.

The differences in LGA prevalence between the reference charts were consistent with previous Swedish findings [30]. The SWE‐REF reference ranges, which yielded the lowest LGA prevalence, excluded women with conditions known to cause abnormal fetal growth but applied no limitations on maternal body mass index (BMI). Eleven per cent of the women were obese (BMI > 30.0 kg/m^2^), which is associated with an increased risk of LGA births [31, 32]. European origin is also associated with higher birthweight [33], and 94.2% of the SWE‐REF study population originated from European countries. This could skew the SWE‐REF ranges toward higher estimated birthweights, leading to underestimation of LGA when used across populations.

The customised GROW calculator, on the other hand, adjusts for maternal height, weight, parity and ethnic origin. It is debated whether these factors, all known to affect fetal growth, have purely physiological effects and should be included in customised growth charts, or whether they may also have pathological effects. A Dutch study showed increasing LGA prevalence with increasing maternal height, using national growth charts. When customising for maternal height, the LGA prevalence was more consistent across maternal heights [10]. However, maternal obesity is globally increasing [34, 35], and adjustment for maternal weight may underestimate LGA in infants of obese mothers. The GROW chart provided LGA proportions closest to the expected 10% (> 90th percentile) and 3% (> 97th percentile).

The Intergrowth‐21st charts are not adjusted for maternal characteristics, although women with BMI < 18.5 or > 30 kg/m^2^ were excluded. Their cohort of healthy, multi‐ethnic women with low‐risk pregnancies is assumed to reflect optimal fetal growth universally. However, data were limited to eight countries, and since ethnic differences in fetal growth are suggested to be physiologic [8], this approach could result in both over‐ and underestimation of LGA. The large proportion of infants classified as LGA using Intergrowth‐21st suggests that it likely overestimates LGA prevalence in this study population. Additionally, Intergrowth‐21st is based on measurements at birth, potentially underestimating growth restriction in preterm births.

Estimated fetal weight (EFW)‐based standards are designed to reflect in utero growth potential in healthy, low‐risk pregnancies, which makes them suitable for classifying birthweight. This is particularly relevant for preterm births, where growth restriction is more common. In such cases, birthweight‐based reference standards may underestimate growth restriction and overestimate LGA. However, EFW‐based standards also have limitations, as they rely on estimated rather than actual weights. Their accuracy is operator‐dependent and can be reduced by factors such as high maternal BMI or fetal size at either end of the spectrum.

Previous studies have shown that GROW better identifies small‐for‐gestational‐age (SGA) infants at risk of adverse neonatal outcomes than Intergrowth‐21st [36, 37, 38]. Similar studies on LGA and adverse neonatal outcomes are limited. In one study, being classified as LGA by both Intergrowth‐21st and GROW was not linked to a higher risk of stillbirth. LGA, as identified by Intergrowth‐21st, alone actually showed a lower relative risk for stillbirth [38]. In studies of offspring of mothers with type 1 diabetes, GROW and Intergrowth‐21st reported similar numbers of LGA and SGA neonates. GROW‐defined LGA correlated slightly better with neonatal complications, whereas the results were the opposite for SGA [39]. In another study performed on a Swedish population, Intergrowth‐21st > 97th percentile corresponded to GROW > 87.6th percentile, and the increased risk of perinatal adverse outcomes occurred at different percentile thresholds. Furthermore, only GROW charts identified an increased risk of perinatal mortality in large infants (> 95th centile) [30]. A European study compared Intergrowth‐21st and national growth charts across 15 countries. LGA prevalence (defined as > 90th percentile) was about 10% in all countries when using their national charts, but varied between 8.0% and 24.9%, with a south‐to‐north gradient, using Intergrowth‐21st [11].

The LGA prevalence variation across different growth charts and percentile thresholds is expected. The main finding of this study is the conflicting interpretations of its results due to different LGA definitions. The results differed both between the reference growth charts and within the same reference chart across percentile thresholds. No change in LGA was observed in the main analyses when defined as > 90th percentile according to the Swedish Maršál et al. growth charts, which was the primary outcome in the CDC4G study. However, in the sensitivity analysis excluding preterm births, LGA was reduced in the subgroup discordant for GDM diagnosis. This is notable, as preterm infants are often growth‐restricted, complicating the interpretation of LGA in this group. This finding may have implications for the interpretation of the CDC4G results.

This secondary analysis demonstrates that if an alternative definition of LGA had been used in the CDC4G study, the incidence of LGA would have decreased following the implementation of the WHO‐2013 GDM criteria. This is both problematic and relevant, as LGA is a recommended [3] and inconsistently defined [4] outcome measure in RCTs, and how results are interpreted can affect clinical practice. However, given the physiological differences in birthweight related to ethnicity [8], a universal definition of LGA may not be preferred. It requires careful definition of the caveats, which may make it too complex for clinical practice. Future studies may explore which growth chart most effectively identifies LGA offspring at increased risk of perinatal morbidity. However, a major challenge remains: results will be influenced by maternal characteristics within the study population, limiting their applicability and generalisability across diverse populations.

## 5 Conclusions

This study highlights the varying definitions of LGA and the importance of interpreting findings in the context of the specific study population and methods used. In future research, establishing consensus to consistently report at least one standardised LGA definition would facilitate comparison across studies. We recommend reporting LGA using both up‐to‐date growth charts used in clinical practice for each study population and customised charts.

## Author Contributions

H.B., S.M., A.M., D.S., K.B., H.S., K.H., U.‐B.W., V.S., F.A., A.‐K.W., E.S.‐L., M.P., S.P. and M.d.B. designed the study and defined the outcomes as part of the CDC4G study protocol. S.P. managed the dataset and A.M. did the statistical analyses. S.G. drafted the manuscript in collaboration with M.d.B and with input from F.A., H.B., M.P. and A.‐K.W. All authors critically reviewed and revised the manuscript and approved the final version for submission.

## Funding

The Swedish Research Council (https://www.vr.se/english.html) (HB), 2018‐00470, ALF Funding Region Örebro County (HB), OLL‐930268, The Swedish state under the agreement between the Swedish government and the county councils, the ALF‐agreement (VS), GBG‐823211, ALFGBG‐932692, Nyckelfonden, Region Örebro County (HB), OLL‐597601, Region Örebro County Research committee (HB), OLL‐693551, OLL‐786911, Regional Research committee Uppsala‐Örebro (HB), RFR‐749241, Stiftelsen Mary von Sydows, född Wijk, donation fund (VS), numbers 1017, 4917, 2618, and 3718, Clinical therapy research, Region Stockholm County, The Centre of Clinical Research (ESL), Västmanland County Council (MdB), LTV‐966501, Research Funds of Skåne University Hospital and the Skåne County Council Research and Development Foundation (KB), REGSKANE‐622891, ALF Funding Region Uppsala, 2024‐19045 (FA). The funders had no role in study design, data collection, analysis, writing of the manuscript or decision to submit the report for publication.

## Conflicts of Interest

The authors declare no conflicts of interest.

## Supporting information


**Table S1:** Baseline characteristics of the modified intention to treat population and the subgroup discordant for diagnosis of GDM*. Already published in the CDC4G main study ^1^.

**Table S2:** Outcomes in sensitivity analyses restricted to term births, i.e., gestational week ≥ 37 + 0.

## Data Availability

The data that support the findings of this study are available on request from the corresponding author. The data are not publicly available due to privacy or ethical restrictions.
