# Supplementary material for: Choice of Growth Standard and Interpretation of Large for Gestational Age Outcomes: Evidence From the CDC4G Trial
Source: Paediatr Perinat Epidemiol. 2026 Jul 29;40(7):785–93. doi: 10.1111/ppe.70180 (PMC13624514; doi:10.1111/ppe.70180)
Supplement: Supplementary file 1 [file PPE-40-785-s001.docx]

|  | mITT population | | | | Subgroup discordant for GDM diagnosis^*^ | | | |
| --- | --- | --- | --- | --- | --- | --- | --- | --- |
|  | **SWE-GDM criteria**  **(n = 22 797)** | | **WHO-2013**  **criteria (n = 24 283)** | | **SWE-GDM criteria**  **(n = 956)** | | **WHO-2013**  **criteria (n = 1 239)** | |
| Maternal characteristics | n |  | n |  | n |  | n |  |
| Age at childbirth, years | 22 797 | 31.6 (28-35) | 24 283 | 31.3 (28-35) | 956 | 32·8 (29-36) | 1 239 | 32.8 (29-36) |
| Body height at first visit, cm | 22 079 | 166 (162-170) | 23 495 | 166 (161-170) | 933 | 166 (162-170) | 1 222 | 165 (160-170) |
| Body weight at first visit, kg | 21 655 | 66 (59-75) | 23 041 | 66 (59-75) | 922 | 86 (70-100) | 1 204 | 83 (69-98) |
| BMI at first visit, kg/m^2^ | 21 591 | 23.7 (21.5-27.0) | 23 041 | 23.9 (21.6-27.3) | 922 | 30.9 (25.6-35.9) | 1 202 | 30.4 (25.6-35.8) |
| Underweight (<18.5) |  | 547 (2.5) |  | 620 (2.7) |  | 5 (0.5) |  | 7 (0.6) |
| Normal (18.5-24·9) |  | 12 705 (58.8) |  | 13 157 (57.1) |  | 192 (20.8) |  | 257 (21.4) |
| Overweight (25.0-29.9) |  | 5 458 (25.3) |  | 5 908 (25.6) |  | 212 (23.0) |  | 300 (25.0) |
| Obesity class I (30.0-34.9) |  | 2 039 (9.4) |  | 2 289 (9.9) |  | 211 (22.9) |  | 273 (22.7) |
| Obesity class II (35.0-39.9) |  | 620 (2.9) |  | 760 (3.3) |  | 219 (23.8) |  | 254 (21.1) |
| Obesity class III (≥40.0) |  | 222 (1.0) |  | 307 (1.3) |  | 83 (9.0) |  | 111 (9.2) |
| Parity^†^ | 22 797 |  | 24 282 |  | 956 |  | 1 239 |  |
| 0 |  | 9 784 (42.9) |  | 9 889 (40.7) |  | 304 (31.8) |  | 370 (30.0) |
| 1 |  | 8 537 (37.4) |  | 9 051 (37.3) |  | 369 (38.6) |  | 463 (37.4) |
| 2 |  | 3 157 (13.8) |  | 3 590 (14.8) |  | 180 (18.8) |  | 235 (19.0) |
| 3 |  | 859 (3.8) |  | 1 114 (4.6) |  | 54 (5.6) |  | 112 (9.0) |
| ≥4 |  | 463 (2.0) |  | 638 (2.6) |  | 49 (5.1) |  | 59 (4.8) |
| Chronic hypertension^‡^ | 22 797 | 182 (0.7) | 24 283 | 202 (0.7) | 956 | 23 (2.4) | 1 239 | 17 (1.4) |
| Smoking at first visit | 21 810 |  | 23 139 |  | 937 |  | 1 206 |  |
| No |  | 21 094 (96.7) |  | 22 309 (96.1) |  | 880 (94.9) |  | 1 144 (94.9) |
| 1-9 cig/day |  | 597 (2.7) |  | 668 (2.9) |  | 37 (4.0) |  | 51 (4.2) |
| ≥10 cig/day |  | 119 (0.6) |  | 162 (0.7) |  | 10 (1.1) |  | 11 (0.9) |
| Swedish snuff at first visit | 22 741 | 191 (0.8) | 24 085 | 236 (1.0) | 955 | 8 (0.8) | 1 232 | 18 (1.5) |
| Country of birth^§^ | 22 790 |  | 24 281 |  | 956 |  | 1 239 |  |
| Sweden |  | 15 592 (68.4) |  | 16 444 (67.7) |  | 588 (61.5) |  | 670 (54.1) |
| Europe except Sweden |  | 2 380 (10.4) |  | 2 459 (10.1) |  | 98 (10.2) |  | 129 (10.4) |
| Middle East and North Africa |  | 2 261 (9.9) |  | 2 494 (10.3) |  | 139 (14.5) |  | 187 (15.1) |
| North America and Caribbean |  | 101 (0.4) |  | 114 (0.5) |  | 3 (0.3) |  | 4 (0.3) |
| South and Central America |  | 303 (1.3) |  | 288 (1.2) |  | 14 (1.5) |  | 16 (1.3) |
| Africa |  | 1 256 (5.5) |  | 1 562 (6.4) |  | 77 (8.0) |  | 135 (10.9) |
| South-East Asia |  | 290 (1.3) |  | 318 (1.3) |  | 18 (1.9) |  | 54 (4.4) |
| Western Pacific |  | 607 (2.7) |  | 602 (2.5) |  | 19 (2.0) |  | 44 (3.5) |
| Highest education, years | 21 982 |  | 23 512 |  | 921 |  | 1 190 |  |
| <9 (school education) |  | 678 (3.1) |  | 858 (3.6) |  | 49 (5.3) |  | 85 (7.1) |
| 9 (school education) |  | 1 231 (5.6) |  | 1 451 (6.2) |  | 65 (7.1) |  | 106 (8.9) |
| 10-11 (school education) |  | 1 530 (7.0) |  | 1 688 (7.2) |  | 79 (8.6) |  | 139 (11.7) |
| 12 (school education) |  | 5 171 (23.5) |  | 5 980 (25.4) |  | 248 (26.9) |  | 308 (25.9) |
| <3 (college/university) |  | 3 455 (15.7) |  | 3 498 (14.9) |  | 145 (15.7) |  | 164 (13.8) |
| ≥3 (college/university) |  | 9 678 (44.0) |  | 9 746 (41.4) |  | 323 (35.1) |  | 378 (31.8) |
| Doctor/licentiate degree |  | 239 (1.1) |  | 291 (1.2) |  | 12 (1.3) |  | 10 (0.8) |
| Neonatal characteristics | 22 738 |  | 24 227 |  | 951 |  | 1 238 |  |
| Boy |  | 11 665 (51.3) |  | 12 328 (51.0) |  | 493 (51.8) |  | 632 (51.0) |
| Girl |  | 11 073 (48.7) |  | 11 899 (49.1) |  | 458 (48.2) |  | 606 (49.0) |

**Table S1:** **Baseline characteristics of the modified intention to treat population and the subgroup discordant for diagnosis of GDM*. Already published in the CDC4G main study ^1^.**

Data are n (%) or median (IQR) unless stated otherwise.

BMI, body mass index; GDM, gestational diabetes mellitus; SD, standard deviation. *The cohort of women with fasting and 2-hour plasma glucose cut off between the WHO-2013 and SWE-GDM criteria (fasting plasma glucose 5·1-6·9 and/or 2-hour plasma glucose 8·5-8.8/8.9/9.9 mmol/L), untreated before and treated after the switch. ^†^Number of previous deliveries; stillbirths or live births.

^‡^Hypertension known before pregnancy or new onset hypertension with blood pressure ≥140/90 mmHg before gestational week 20. ^§^Grouped according to International Diabetes Federation Atlas except for having an extra category for Sweden.

1.	de Brun M, Magnuson A, Montgomery S, Patil S, Simmons D, Berntorp K, et al. Changing diagnostic criteria for gestational diabetes (CDC4G) in Sweden: A stepped wedge cluster randomised trial. PLoS Med. 2024;21(7):e1004420.
