# Supplementary material for: Choice of Growth Standard and Interpretation of Large for Gestational Age Outcomes: Evidence From the CDC4G Trial
Source: Paediatr Perinat Epidemiol. 2026 Jul 29;40(7):785–93. doi: 10.1111/ppe.70180 (PMC13624514; doi:10.1111/ppe.70180)
Supplement: Supplementary file 2 [file PPE-40-785-s002.docx]

| Modified intention to treat | | | | | | |
| --- | --- | --- | --- | --- | --- | --- |
|  | **WHO-2013 GDM**  **criteria group**  **(n = 23 139)** | | **SWE-GDM**  **criteria group**  **(n = 21 854)** | | **WHO-2013 vs. SWE-GDM criteria group** | |
|  |  |  |  |  | Adjusted 1^b^ | Adjusted 2^c^ |
| LGA | n | Outcomes (%) | n | Outcomes (%) | RR (95% CI) | RR (95% CI) |
| LGA 90^th^ perc SWE REF | 23,100 | 1 222 (5.3) | 21,800 | 1 199 (5.5) | 0.88 (0.82-0.94) | 0.89 (0.84-0.94) |
| LGA 90^th^ perc, GROW | 23,094 | 2 155 (9.3) | 21,797 | 2 133 (9.8) | 0.96 (0.90-1.01) | 0.95 (0.89-1.01) |
| LGA 90^th^ perc, Intergrowth-21^st^ | 23,098 | 5 755 (24.9) | 21,798 | 5 446 (25.0) | 0.99 (0.94-1.03) | 0.99 (0.94-1.04) |
| Severe LGA |  |  |  |  |  |  |
| LGA 97^th^ perc, SWE REF | 23,100 | 360 (1.6) | 21,800 | 338 (1.6) | 0.87 (0.77-0.99) | 0.88 (0.76-1.01) |
| LGA 97^th^ perc, GROW | 23,094 | 836 (3.6) | 21,797 | 800 (3.7) | 0.94 (0.87-1.02) | 0.93 (0.84-1.03) |
| LGA 97^th^ perc, Intergrowth-21^st^ | 23,098 | 2 238 (9.7) | 21,798 | 2 126 (9.8) | 0.93 (0.87-0.99) | 0.93 (0.86-1.01) |
| Results from the CDC4G main study^d^ |  |  |  |  |  |  |
| LGA 90^th^ perc, Marsal et al | 23,100 | 2 657 (11.5) | 21,800 | 2 515 (11.5) | 0.95 (0.90-0.99) | 0.95 (0.89-1.02) |
| LGA 2 SD, Marsal et al | 23,100 | 1 005 (4.4) | 21,800 | 983 (4.5) | 0.88 (0.81-0.96) | 0.89 (0.83-0.95) |
|  |  |  |  |  |  |  |
| Subgroup discordant for GDM diagnosis^a^ | | | | | | |
|  | **WHO-2013 GDM**  **criteria group**  **(n = 1 163)** | | **SWE-GDM**  **criteria group**  **(n = 917)** | | **WHO-2013 vs. SWE-GDM criteria group** | |
|  |  |  |  |  | Adjusted 1^b^ | Adjusted 2^c^ |
| LGA | n | Outcomes (%) | n | Outcomes (%) | RR (95% CI) | RR (95% CI) |
| LGA 90^th^ perc, SWE REF | 1 162 | 150 (12.9) | 911 | 180 (19.8) | 0.56 (0.43-0.74) | 0.64 (0.48-0.86) |
| LGA 90^th^ perc, GROW | 1 162 | 171 (14.7) | 910 | 201 (22.1) | 0.76 (0.60-0.96) | 0.75 (0.61-0.92) |
| LGA 90^th^ perc, Intergrowth-21^st^ | 1 162 | 422 (36.3) | 911 | 412 (45.2) | 0.83 (0.68-1.03) | 0.86 (0.72-1.05) |
| Severe LGA |  |  |  |  |  |  |
| LGA 97^th^ perc, SWE REF | 1 162 | 64 (5.5) | 911 | 59 (6.5) | 0.75 (0.42-1.33) | 0.77 (0.39-1.50) |
| LGA 97^th^ perc, GROW | 1 162 | 89 (7.7) | 910 | 98 (10.8) | 0.92 (0.55-1.54) | 0.86 (0.56-1.31) |
| LGA 97^th^ perc, Intergrowth-21^st^ | 1 162 | 206 (17.7) | 911 | 236 (25.9) | 0.69 (0.60-0.80) | 0.73 (0.64-0.84) |
| Results from the CDC4G main study^d^ |  |  |  |  |  |  |
| LGA 90^th^ perc, Marsal et al | 1 162 | 264 (22.7) | 911 | 267 (29.3) | 0.81 (0.69-0.95) | 0.85 (0.74-0.98) |
| LGA 2 SD, Marsal et al | 1 162 | 132 (11.4) | 911 | 153 (16.8) | 0.70 (0.47-1.03) | 0.76 (0.62-0.94) |
|  |  |  |  |  |  |  |

**Table S2. Outcomes in sensitivity analyses restricted to term births, i.e. gestational week ≥ 37+0.**

^a^ The cohort of women with fasting and 2-hour plasma glucose cut off between the WHO-2013 criteria and SWE-GDM criteria (fasting plasma glucose 5.1-6.9 and/or 2-hour plasma glucose 8.5-8.8/8.9/9.9 mmol/L), untreated before and treated after the switch. ^b^ Analysed with multilevel mixed Poisson model with cluster as random factor and period (January-March, April-June, July-September, October-December) as fixed factor. ^c^ Adjusted also for mother’s age modelled by a linear, squared and cubic term, chronic hypertension, smoking, snuff, country of birth and parity and multiple imputation used for missing potential confounding variables. ^d^Results from the CDC4G main study, included for comparison ^12^

RR, Relative risk; CI, Confidence interval; Perc, Percentile; GDM, gestational diabetes mellitus.
